# From Liking to Following: The Role of Food Preferences, Taste Perception, and Lifestyle Factors in Adherence to the Mediterranean Diet Among Young Individuals

**DOI:** 10.3390/nu17030600

**Published:** 2025-02-06

**Authors:** José V. Sorlí, Edurne de la Cámara, José I. González, Olga Portolés, Ignacio M. Giménez-Alba, Rebeca Fernández-Carrión, Oscar Coltell, Inmaculada González-Monje, Carmen Saiz, Eva C. Pascual, Laura V. Villamil, Dolores Corella, Eva M. Asensio, Carolina Ortega-Azorín

**Affiliations:** 1 Department of Preventive Medicine and Public Health, School of Medicine, University of Valencia, 46010 Valencia, Spain; doctora@delacamara.es (E.d.l.C.); ignacio.glez-arraez@uv.es (J.I.G.); olga.portoles@uv.es (O.P.); i.gimenez.alba@uv.es (I.M.G.-A.); rebeca.fernandez@uv.es (R.F.-C.); inmaagonzalez@gmail.com (I.G.-M.); carmen.saiz@uv.es (C.S.); pascaseva89@gmail.com (E.C.P.); laura.v.villamil@uv.es (L.V.V.); eva.m.asensio@uv.es (E.M.A.); carolina.ortega@uv.es (C.O.-A.); 2 CIBER Fisiopatología de la Obesidad y Nutrición, Instituto de Salud Carlos III, 28029 Madrid, Spain; oscar.coltell@uji.es; 3 Servicio de Oftalmología, Hospital Clínico Universitario Lozano Blesa, 50009 Zaragoza, Spain; 4 Department of Computer Languages and Systems, Universitat Jaume I, 12071 Castellón, Spain; 5 Department of Physiology, School of Medicine, University Antonio Nariño, Bogotá 111511, Colombia

**Keywords:** dietary patterns, lifestyles, overweight, obesity, Mediterranean diet, food preferences, taste, cardiovascular risk, young individuals

## Abstract

Background and aims: The Mediterranean diet (MedDiet) is a healthy dietary pattern associated with reduced risk of chronic diseases. However, adherence is declining, particularly among younger populations. Therefore, it is crucial to identify the main aspects that affect its adherence, particularly food preferences and sensory function, which have received insufficient attention. Our aims were to investigate the impact of socio-demographic and lifestyle factors on adherence to the MedDiet among young individuals; to assess the association of taste preferences and food liking with MedDiet adherence; and to evaluate the associations between taste perception modalities, taste and food preferences, and adherence. Methods: A cross-sectional study on young adults (aged 20.5 ± 4.7 years) in a Mediterranean country (n = 879) was carried out. Demographic characteristics, clinical characteristics, anthropometric characteristics, lifestyle characteristics, MedDiet adherence, taste preferences, and food preferences were assessed. Taste perception for bitter, salty, sweet, sour, and umami was determined by rating different concentrations of prototypical tastants. We computed a total taste perception score and three scores to combine food preferences. Results: We identified several socio-demographic and lifestyle factors associated with adherence to the MedDiet, as well as food preferences, taste preferences, and taste perception determinants. Food preferences were significantly associated with total adherence to the MedDiet. Higher preference for olive oil, oranges, broccoli, fish, and legumes was associated with higher MedDiet adherence (*p* < 0.05 for all). Conversely, higher preference for sweet foods, red meat, and butter was associated with lower adherence (*p* < 0.05 for all). The combined positive score for food preference was strongly associated with higher MedDiet adherence (*p* = 1.4 × 10^−23^) in the multivariate adjusted model. The combined negative food preference score was inversely associated (*p* = 1.9 × 10^−8^). Likewise, taste preferences were significantly associated with adherence to the MedDiet (strong inverse association for sweet taste preference and direct association for bitter taste preference; both *p* < 0.001). Moreover, bitter taste perception was inversely associated with adherence to the MedDiet and with bitter foods (*p* < 0.05). In conclusion, future precision nutrition studies should measure food liking and taste preferences, which are crucial determinants of MedDiet adherence, especially in young people.

## 1. Introduction

Noncommunicable diseases, also known as chronic diseases, are the leading causes of death worldwide, and several key drivers of these diseases have been identified. Obesity is a relevant risk factor for a variety of chronic diseases, such as type 2 diabetes, hypertension, dyslipidemias, cardiovascular diseases, certain types of cancer, respiratory diseases, renal and liver diseases, cognitive decline, and mental health-related disorders, among others [1,2,3,4,5,6,7,8,9,10,11,12,13,14,15,16,17]. Moreover, although prevalence rates of obesity vary widely among countries, obesity has increased in prevalence worldwide across all age categories and sexes, according to research on global obesity trends [18,19,20,21]. Considering the serious risks related to comorbidities, these increasing trends in prevalence necessitate collaborative efforts from the scientific community, individual patients, and governments at all levels to mitigate the obesity pandemic and to reduce the impact of comorbidities. Obesity is a complex condition influenced by both genetic and environmental factors [22,23,24]. Therefore, public health prevention strategies, developed in many countries based on traditional nutrition and physical activity promotion, have not achieved substantial results. Regarding nutrition, in recent years, it has been suggested that precision (or personalized) nutrition, which promotes individualized needs over the formulation of general recommendations for the population, has the potential to enhance nutritional objectives [25,26,27,28]. Although precision nutrition is still in the early stages of knowledge generation, it is hypothesized that discoveries involving new omics biomarkers or the identification of relevant individual characteristics influencing dietary behaviors could be applied to a novel public health field known as Precision Public Health [29,30]. Precision Public Health relies on more precise and higher-quality data to target disease prevention and control in the appropriate population at the right time [30,31,32]. Therefore, in order to more accurately estimate the dietary requirements of different subgroups and to facilitate more effective dietary recommendations and interventions, additional data addressing the individual characteristics that are associated with a different nutritional response or low adherence to dietary patterns are essential.

In terms of dietary patterns that are most closely associated with healthful eating and exhibit an important inverse association with obesity, the Mediterranean diet (MedDiet) stands out [33,34,35,36,37,38,39]. A reduced incidence of cardiovascular diseases, type 2 diabetes, chronic kidney disease, fatty liver disease, cognitive decline, and other diseases has also been associated with the MedDiet [40,41,42,43,44,45,46,47,48,49]. Nonetheless, despite the numerous health benefits associated with the MedDiet, compliance among younger individuals in Mediterranean countries is decreasing [50,51,52,53,54,55,56,57,58,59], and adherence to the traditional MedDiet remains low in non-Mediterranean countries as well [60,61]. Consequently, from a public health point of view, increasing adherence to the MedDiet is crucial for mitigating the risk of numerous chronic diseases; thus, it is imperative to have a deeper understanding of the factors influencing adherence to this dietary pattern. Although some researchers have examined MedDiet adherence determinants, most have simply examined socio-demographic and lifestyle variables. Thus, in addition to analyzing the influence of age, associations with sex, socio-economic status, physical activity, tobacco smoking, alcohol consumption, food consumption, sleep habits, and other social determinants or health conditions have been investigated [51,52,53,54,55,56,57,58,59,60,61,62,63,64,65,66,67,68,69,70]. However, there are scarcely any studies that have examined the impact of preferences for foods associated with the MedDiet, or taste perception and other gustatory functions, on the degree of adherence to the MedDiet pattern [71,72,73,74,75,76,77]. Thus, Louro et al. [71] analyzed the association of taste perception and preference for four tastes (sweet, sour, salty, and bitter) with adherence to the MedDiet in 383 adults from Portugal, and found that the most relevant association of adherence was with aromatic plant intake. Likewise, in 400 adults from Australia, a moderate to high adherence to the MedDiet was associated with daily use of herbs and spices [73]. Other research has focused on how salt taste perception [72], sweet taste preference [74], or olive oil preference [75] are related to MedDiet adherence or to the consumption of some specific foods. Furthermore, none of these studies [71,72,73,74,75,76,77] conducted an in-depth investigation of the associations of individuals’ taste preferences, taste perceptions, and food preferences with their adherence to the MedDiet and its specific food components.

While there are some variations that are dependent on the country, the traditional MedDiet is generally considered to be characterized by the following: a high consumption of vegetables, fruits, legumes, cereals, nuts, and olive oil; a moderate to high fish consumption; a minimal consumption of red meats and meat products; a moderate consumption of poultry and dairy products; and a moderate red wine intake [78,79]. See Supplementary Table S1 for more details of the foods and servings included in the traditional MedDiet. The inclusion of red wine in the MedDiet definition is controversial, due to disagreements about the negative aspects of alcohol [80,81]. Previous research that has not been specifically directed toward the MedDiet has demonstrated that food and taste preferences are highly associated with food intake [82,83,84,85,86,87,88]. In general, people eat the food they like. Food preferences have been described as “an individual’s reported degree of liking for specific foods and beverages without regard to food intake per se” [85]. Despite the relevance of food preference and taste preference in determining food intake [82,83,84,85,86,87,88,89,90], the vast majority of large studies analyzing adherence to the MedDiet have not employed food preference questionnaires, resulting in an unknown understanding of these associations. Additionally, it is necessary to examine the factors that exert an influence on food preferences. One of the potentially most influential factors, in addition to socio-demographic variables, is taste perception [91,92,93,94,95,96]. It is well established that the capacity to perceive tastes is highly variable, and genetic factors significantly influence this perception, particularly in the case of bitterness [96,97,98,99,100]. In addition, it has been hypothesized that an increased capacity to detect a particular taste modality is related to a decreased preference for that taste and a decrease in the consumption of foods that are rich in that taste modality, but results are inconsistent [98,101,102,103,104,105,106,107]. Despite the fact that there are sensory tests available in the laboratory to detect the ability to perceive the different basic tastes (sweet, salty, bitter, sour, and umami) [98,108,109], these tests are laborious and have not been conducted in large nutritional epidemiology cohorts, making their integrated study necessary. Thus, due to the lack of this integrated knowledge, our objectives are as follows: (1) to examine the influence of socio-demographic and lifestyle variables on adherence to the MedDiet among young individuals from a Mediterranean population; (2) to estimate the association of taste preferences and food liking with adherence to the MedDiet; and (3) to analyze the association of taste perception modalities with taste and food preferences and adherence to the MedDiet, taking into account other taste-, socio-demographic-, and lifestyle-related factors.

## 2. Materials and Methods

### 2.1. Study Design and Participants

We conducted a cross-sectional study at the University of Valencia, Spain, in order to analyze mainly young participants from a Mediterranean country with a similar educational level, to minimize inter-individual differences in potential confounding factors. Students were recruited from the School of Medicine, University of Valencia. Second-year undergraduate medical students were eligible for participation, as they were granted access to the sensory test laboratory at the School of Medicine. Students were informed about the study and were invited to participate in class by researchers involved in this investigation. The exclusion criteria included any allergy to the prototypical tastants utilized in the taste perception tests; acute manifestations of respiratory, digestive, renal, or hepatic diseases; pregnancy or lactation; debilitating physical or psychological disorders; a cancer diagnosis; thyroid abnormalities; Cushing’s disease; the presence of any infectious or contagious disease; excessive alcohol consumption; or the use of other drugs. The recruitment started in 2015 and finished in 2018. A total of 879 participants (84% response rate) including both men and women (264 and 615, respectively) were analyzed in this study. The average age was 20.5 years, with a standard deviation of 4.7 years. Students were from the Valencia region, which is located on the East Mediterranean coast of Spain, as well as from other Spanish regions and international locations. Based on the sample size calculations from our previous study on taste perception associations, a power of over 80% was achieved at a 5% alpha error by including a minimum of 381 participants [97]. By assessing 879 participants, the statistical power was enhanced, enabling the carrying out of additional stratified analyses. Included participants completed questionnaires, as well as taste perception tests, in the laboratory. Participants were apparently healthy individuals, and provided written informed consent. The study was undertaken at the Department of Preventive Medicine and Public Health, School of Medicine at the University of Valencia, Valencia. The study protocols and procedures were approved according to the ethical standards of the Helsinki Declaration by The Ethics Committee of Research in Humans of the Ethics Commission in Experimental Research of the University of Valencia, Valencia (approval codes: H1418906866769, 3 February 2015; CE1575538495119, 5 December 2019; and CE2208970, 13 September 2022).

### 2.2. Demographic, Clinical, Anthropometric, and Lifestyle Variables

A general questionnaire assessed socio-demographic data (sex, age, geographical origin), lifestyle factors (smoking, drinking, physical activity, and sleep duration), and other factors, as previously reported [98]. We evaluated both current and former smoking status. Current smokers were defined as those smoking at least one cigarette a day. Former smokers were defined as those who smoked regularly—at least one cigarette per day—but had not smoked for over a month before the examination [110]. A set of 22 questions evaluated alcohol intake during workdays and weekends, further categorizing drinkers into non-drinkers and those who consumed any amount of alcohol [110]. Physical activity was estimated from questions about daily walking for at least 20 min, as well as about practicing regular physical exercise in leisure time, as previously reported [111]. We created two dichotomous variables for walking and physical exercise. We assessed weight and height directly using calibrated scales and a wall-mounted stadiometer, respectively, adhering to a standard protocol [98]. We determined individuals’ body mass index (BMI) by dividing their weight in kilograms by the square of their height in meters. We used a validated semiautomatic oscillometer (Omron HEM-705CP, ‘s-Hertogenbosch (Den Bosch), Netherlands) to measure blood pressure (systolic and diastolic) and resting heart rate twice, while the individual remained seated for 5 min, as previously reported [98].

### 2.3. Adherence to MedDiet, Food Preference, and Taste Preference Assessment

To measure adherence to the MedDiet pattern, we used the 14-item MEDAS scale validated by us in the PREDIMED study [112]. Supplementary Table S1 presents the detailed items of that scale, along with their response options. We scored each question either 0 or 1. The final score ranged from 0 to 14. The higher the score, the greater the adherence to the MedDiet. The degree of adherence was later dichotomized into high (≥9) and low (<9), depending on the population mean (approximately 9 points). In addition, a three-category variable was created: low adherence (from zero to 7), intermediate adherence (8 and 9 points), and high adherence (from 10 to 14 points).

We used a food preference questionnaire to assess the degree of preference for different foods based on a Likert-type 4-point scale, as previously reported [74]. We used this simpler 4-point Likert scale by adapting the 9-point scale of previous food linking questionnaires [113,114], in order to simplify the selection process. We removed the mid-point category (neither like nor dislike) to minimize neutral position bias [85,115]. Therefore, we asked the participants about their preferences for selected foods. We obtained responses using a 4-point Likert scale, which scored food preferences on an increasing scale (0, 1, 2, and 3) of “strongly dislike”, “dislike”, “like”, and “strongly like”. We repeated the same responses to gather data on preferences for 37 food items included in the questionnaire. These foods were selected based on the MedDiet, as well as on the five basic taste qualities. The questionnaire included the following food items: whole milk, semi/skimmed milk, whole yogurt, semi/skimmed yogurt, eggs, red meat, poultry, blue fish, white fish, seafood, mature cheeses, fresh cheese, red meats, ham and sausages, bread, pasta, legumes, French beans, broccoli, artichokes, oranges, lemons, other fruits, olive oil, sunflower oil, other oils, butter, margarine, aioli, mayonnaise, salt-cured foods, spicy foods, spices, nuts, breakfast cereals, pastries/ice cream, chocolates, and sugar, as previously reported [74]. We analyzed the preference for these foods (ranging from zero to three) both individually and by calculating the combined scores of the foods under consideration. We computed a so-called positive score for food preference as the sum of the ratings of the selected foods, including olive oil, nuts, white fish (as a proxy for fish), broccoli (as a proxy for vegetables), oranges (as a proxy for fruits), and legumes, as these food items were included in the 14-item MedDiet score, and had a direct rating. Likewise, we computed a negative score for food preference as the sum of the ratings of the selected foods included in the MedDiet scale with inverse association (red meats, pastries, and butter). Further, we reversed the score of the negatively associated food items, and calculated the total preference food score as the sum of the positive score and the reversed negative score. The preference for different tastes (sweet, salty, bitter, and sour) was measured using a similar 4-point hedonic scale, as previously reported [98], ranging from “strongly dislike” to “strongly like”. No stimuli for taste preference were administered.

### 2.4. Taste Perception Tests

Taste perception tests were carried out on the 879 participants using the same methodology as previously reported by our group for the Mediterranean population [97,98]. More information regarding the selected tastants, concentrations, validity, and potential bias can be obtained from our previous research [97,98]. Briefly, in our laboratory, we conducted taste perception tests in the morning. Trained staff provided a detailed explanation of the procedures prior to starting the series of tests. We selected a representative compound for each taste quality (bitter, sweet, salty, sour, and umami) and administered it at five different concentrations. We used the following tastants (all from Sigma-Aldrich, Milan, Italy) for each taste: 6-n-propylthiouracil (PROP), sucrose, NaCl, citric acid, and L-glutamic acid monopotassium salt monohydrate (MPG) for bitter, sweet, salty, sour, and umami tastes, respectively. We used distilled water as the solvent. Each tastant was presented to the subjects independently. The various solutions of different concentrations were prepared for each tastant by trained personnel, also including a distilled water control. The series of concentrations (concentrations I, II, III, IV, and V, respectively) used for each tastant was based on previous reports [97,98,116,117], and the concentrations were as follows: for PROP (0.055, 0.17, 0.55, 1.7, and 5.5 mM); for sucrose (100 mM, 150 mM, 200 mM, 300 mM, and 400 mM), for NaCl (25 mM, 50 mM, 75 mM, 100 mM, and 200 mM); for citric acid (1 mM, 5 mM, 10 mM, 17 mM, and 34 mM); and for MPG (25 mM, 50 mM, 75 mM, 100 mM, and 200 mM). Bitter taste perception tests (PROP) were undertaken on strips of filter paper, as previously reported [98]. We prepared and tested the other tastants in liquid form, dissolved them to the indicated concentration, and presented them in different-colored small tubes for each taste. Before beginning the taste perception tests, participants had to rinse their mouths several times with spring water. We gave all participants a template on which they had to rate the intensity of each taste and concentration on a scale. The scale consisted of 6 intensity values (from 0 to 5), with 0 meaning “no taste” and 5 meaning “extremely strong.” Subjects rated the corresponding tastant solution or strip, as previously reported [97,98]. We used the same scoring scale for all tastes. Finally, with the scores for the individual taste qualities for concentration V, we constructed a “total taste score”, summing up the points obtained for each of the individual tastes for the concentration tested. The range of the total taste score for each concentration was from 0 to 25 points. In this study, we selected the highest concentration (concentration V) for the associations between food preference and adherence to the MedDiet, in order to maximize the differences in intensity rating between individuals. A higher score indicated a higher taste perception.

### 2.5. Statistical Analysis

Descriptive analyses evaluated the distribution of the variables. We used chi-square tests to analyze proportions, and Student’s *t*-tests and ANOVA tests were used to compare crude means of continuous variables. Pearson or Spearman correlation coefficients were estimated for the corresponding bivariate associations. Three age groups were considered, and several analyses were stratified by age group or by sex. In addition to the original variable, several composite scores were computed, as previously described (including the positive, negative, and total food preference scores, as well as the total taste score for taste perception). We analyzed the associations between the variable of interest (socio-demographic, lifestyle, food preference, taste preference, taste perception, or the composite scores) and the dependent variable of adherence to the MedDiet (as continuous or as categorical, depending on the model) using multivariable regression models, sequentially adjusted for potential confounders as follows: Model 1 was unadjusted; Model 2 added adjustment for sex, age, and geographical origin; whereas Model 3 added adjustment for BMI, smoking, drinking, physical activity, and sleep duration. Additional mutual adjustment for taste preferences or food preference scores was carried out as detailed in the Results Section. We used general linear models for continuous variables as the dependent variable. Logistic regression models were used for dichotomous variables (low or high MedDiet adherence, or compliance with one specific food item). When indicated, we estimated the adjusted means for the continuous variables from the corresponding multivariate corrected models. We performed statistical analyses using IBM SPSS Statistics (version 26.0, New York, NY, USA) and specific tools in R, or using GraphPad Prism 10 for Windows 64-bit (version 10.4.1. Boston, MA, USA). All tests were two-tailed, and *p*-values < 0.05 were considered statistically significant for associations.

## 3. Results

### 3.1. General Characteristics of Participants

Table 1 shows the demographic, clinical, and lifestyle characteristics and adherence to the MedDiet of the study participants (n = 879) by sex (n = 264 men and 615 women).

All of the participants were students of medicine at the University of Valencia (second-year), and the mean age was 20.5 ± (4.7 SD) years, with slight differences by sex (21.3 years in men and 20.1 years in women; *p* < 0.001). We considered three age groups (18–19 years; 20–29 years; and ≥30 years). Age ranged from 18 to 59, but the majority of participants were in the 18–19 years age group (n = 668; 76%), followed by the 20–29 years age group (n = 173; 19.7%). The participants’ geographical origin was mainly the Valencia region (n = 701; 79.7%), followed by other regions of Spain (n = 138; 15.7%). In addition, 4.6% (n = 40) of international students participated in the research. These students primarily came from Europe (n = 18) and America (n = 15), with a smaller minority from Africa (n = 3), Asia (n = 3), and Oceania (n = 1).

The prevalence of obesity was minimal (1.1%), and the prevalence of overweight was only 9.4% in the whole population. Nevertheless, we identified substantial disparities by sex (20.1% in men versus 4.5% in women; *p* < 0.001). Additionally, we identified 10.5% of the population as underweight (BMI < 18.5 kg/m^2^). Significant sex differences were observed (13.7% in women and 3.0% in men; *p* < 0.001). The prevalence of current smokers was 6.3%, being higher in men (*p* = 0.023). We did not detect significant differences by sex in terms of former smokers, sleep duration, alcohol non-drinkers, or mean adherence to the MedDiet (*p* > 0.05). Physical activity was higher in men than in women (*p* < 0.05).

### 3.2. Association of Socio-Demographic and Lifestyle Variables with MedDiet Adherence

In the univariate analysis (Table 1), we did not observe statistically significant differences in global adherence to the MedDiet by sex (*p* = 0.746 for the mean comparisons). However, when we analyzed compliance with the MedDiet recommendations for each of the 14 items, we found some statistically significant differences by sex (Table 2).

Compliance with a high vegetable consumption, as well as with a high intake of nuts, was statistically higher in women (*p* = 0.003). However, men had a higher intake of red meats, and their compliance with the recommendations for this item was low in comparison to women (*p* < 0.001). Intake of the recommended amounts of olive oil was also higher in men (*p* < 0.001). One man and one woman had missing data in the response to a food item; thus, the total score for the global adherence was not calculated for these individuals.

Further, we analyzed, in a multivariate regression model, the association of socio-demographic and lifestyle variables with global adherence to the MedDiet (as continuous). We observed a strong association with age, with adherence increasing as age (in years) increased (B = 0.048; SE: 0.015; *p* = 0.002). In terms of geographical origin, we did not detect significant differences between participants from the Valencia region and those from other Spanish regions (*p* > 0.05), but we observed statistically significant differences in the comparison between Spanish participants and international students, with adherence being higher in Spanish participants (B = 0.713; SE: 0.318; *p* = 0.023). In this multivariate model, adherence to the MedDiet was higher in women (B = 0.243; SE: 0.153), but did not reach statistical significance (*p* = 0.112). No significant associations were found between adherence and sleep duration (*p* = 0.357), current smokers (*p* = 0.740), drinkers (*p* = 0.909), or BMI (*p* = 0.588). However, we detected direct and statistically significant associations of adherence to the MedDiet with the two variables indicating higher physical activity (B = 0.595; SE: 0.211; *p* = 0.005 for people walking more than 20 min per day; B = 0.426; SE: 0.148; *p* = 0.004 for participants practicing at least one form of physical exercise).

### 3.3. Association Between Food Preferences and Adherence to the MedDiet

We used the food preference questionnaire described in the Methods Section to assess the degree of preference for different foods (37 items), based on an increasing Likert-type four-point scale. The higher the score, the higher the preference/liking for the food item. Table 3 shows the Spearman correlation coefficients between each selected food item (n = 32) and the global adherence to the MedDiet in the whole population. We observed positive and highly statistically significant (*p* < 0.001) associations between greater preference for vegetables (broccoli, r = 0.24; artichokes, r = 0.22; French beans, r = 0.24) and greater adherence to the MedDiet. Likewise, greater preference for fish (white fish, r = 0.20; blue fish, r = 0.21), fruits (oranges, r = 0.22; other fruits, r = 0.17), legumes (r = 0.22), nuts (r = 0.12), and olive oil (r = 0.13) was directly and significantly (*p* < 0.001) associated with higher adherence to the MedDiet. Conversely, a higher preference for red meats (r = −0.16), pastries (r= −0.13), and butter (r = −0.11) was inversely associated (*p* ≤ 0.001) with higher adherence to the MedDiet. All of these associations remained statistically significant after fully multivariable adjustment in Model 3.

In addition to the 32 food items presented in Table 3, the other 5 food items included in the questionnaire were aioli, mayonnaise, salt-cured foods, spices (oregano, thyme, bay leaves), and spicy food. We only obtained statistically significant associations of adherence to the MedDiet with spices (r = 0.16; *p* < 0.001) and mayonnaise (r = −0.10; *p* = 0.003). Supplementary Table S2 shows the percentages for each preference category by sex, as well as the statistical significance of each rating by sex. Women showed a higher preference for broccoli and artichokes compared to men (*p* = 0.024 and *p* = 0.029, respectively). Conversely, the preference for red meats (*p* < 0.001) and ham/sausages (*p* = 0.008) was higher in men than in women. All of these associations remained statistically significant after fully multivariable adjustment in Model 3.

After examining the associations between the preference for individual foods and global adherence to the MedDiet, we calculated composite scores for several foods using the ratings from the four-item Likert scale. Therefore, as detailed in the Methods section, we calculated the so-called “positive score” for food preference, which was the total rating of the selected foods that had positive correlation coefficients with adherence to MedDiet. When analyzing multiple foods, we selected only one from each group. For example, we selected the item “broccoli” as a proxy for vegetables, “white fish” as a proxy for fish, and “oranges” as a proxy for fruits; to these foods, we added preference for legumes and preference for olive oil. We chose these foods solely due to their inclusion in the 14-item scale’s compliance. Supplementary Figure S1 shows the frequency distribution of the composite food preference “positive” score (panel A). Likewise, we computed the “negative” score for food preference as the sum of the ratings of the selected foods included in the MedDiet scale with inverse association (red meats, pastries, and butter). Further, we reversed the score of the negatively associated food items, and calculated the “total” preference food score as the sum of the positive score and the reversed negative score. Supplementary Figure S1 also shows the frequency distribution of the “negative” score (panel B) and of the “total” score (panel C).

Further, we tested the associations between the food preference scores (positive, negative, and total) and global adherence to the MedDiet. We obtained very high statistically significant associations for the three variables in the whole population (Table 4).

**Figure 1:**
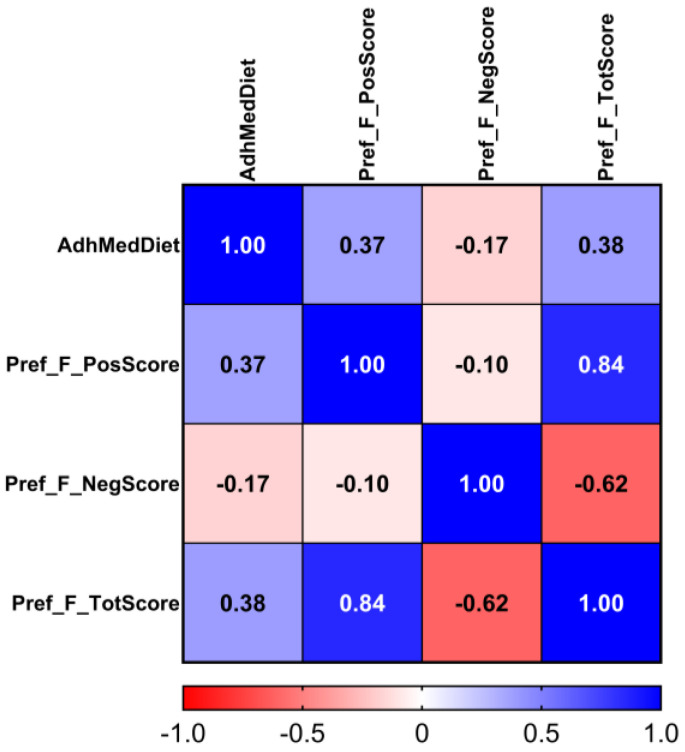
Heatmap for correlation between adherence to Mediterranean diet and food preference scores (positive, negative, and total) in whole population (n = 877). Color intensity is proportional to Pearson correlation coefficients (see color bar for details: warm red colors represent negative correlations, and cool blue colors denote positive correlations). Annotations within squares indicate exact correlation (Pearson) coefficient between each pair of variables. Coefficients ≥ 0.10 are statistically significant (*p* < 0.01).

Participants in the category of high adherence to the MedDiet (from 10 to 14 points) had a higher combined food preference for the above-mentioned foods integrated into the positive preference food score. The association was very high in Model 1, and remained statistically significant in Model 2 and Model 3 after the fully multivariate adjustment (*p* = 1.37 × 10^−23^). For the negative food preference score, the association was inverse. We observed a higher preference for the foods included in the negative score when adherence to the MedDiet was low (from 1 to 7). This association remained statistically significant after the fully multivariate adjustment in Model 3 (*p* = 1.99 × 10^−8^). Finally, we reversed the “negative” food preference score and added the results to the positive score. As expected, the so-called “Total” food preference score was more strongly associated with global MedDiet adherence than the other food preference scores (*p* = 4.6 × 10^−29^ in the fully adjusted Model 3). Moreover, we obtained the Pearson correlation coefficients among the three variables to test for a correlation between the positive and negative scores. Figure 1 shows the heatmap of the correlation coefficients between continuous adherence to the MedDiet, positive food preference score (r = 0.37; *p* = 5.8 × 10^−29^), negative food preference score (r = −0.17; *p* = 2.3 × 10^−7^), and total food preference score (r = 0.38; *p* = 6.1 × 10^−32^) in the whole population.

We obtained very high statistically significant correlations of the positive and total food preference scores with adherence to the MedDiet, and constated that the positive and negative food preference scores were also correlated with each other (r = −0.10; *p* = 0.004).

**Figure 2:**
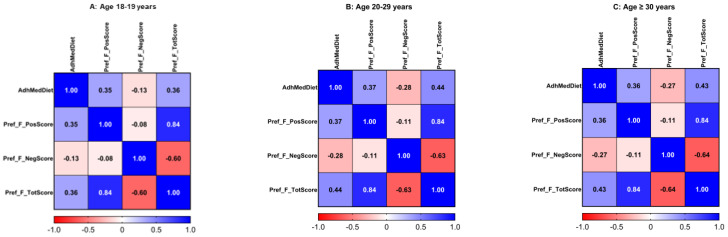
Heatmaps for correlation between adherence to Mediterranean diet and food preference scores by age group ((**A**): 18–19 years; (**B**): 20–29 years; and (**C**): ≥ 30 years). Values are Pearson correlation coefficients ((**A**): n = 668; (**B**): n = 173; (**C**): n = 38). For panel A, all correlation coefficients are statistically significant at *p* < 0.05.

Additionally, we tested whether the correlations among the food preference scores and adherence to the MedDiet were different depending on the age group. Figure 2 shows heatmaps for the same correlation depicted in Figure 1, but stratified by the three age groups (Panel A: subjects aged 18–19 years; Panel B: 20–29 years; and Panel C: 30 years and older). In general, we observed similar correlations between these variables in the three age groups. Statistically significant associations were higher in the group aged 18–19, taking into account the largest sample size of this group in comparison with the others. However, the correlation coefficients were similar or a little bit higher in the 20–29 and ≥30 age groups. Thus, the Pearson correlation coefficient between adherence to the MedDiet and the total food preference score was r= 0.36, *p* = 3.2 × 10^−21^ for participants aged 18–19 years; r = 0.44, *p* = 1.2 × 10^−9^ for participants aged 20–29 years; and r = 0.43; *p* = 0.007 for participants aged ≥ 30 years. This analysis yields a highly relevant result.

Moreover, we analyzed the associations between the food preference scores (positive, negative, and total) and adherence to the MedDiet, which were similar depending on geographical origin: Valencia, other Spanish regions, and international. Supplementary Figure S2 shows the corresponding heatmaps of the Pearson correlation coefficients in Panel A, Panel B, and Panel C, respectively. For subjects from Spain (Panel A versus Panel B), we detected very similar results. The results were the same for both Spanish and international students, with the only difference being that the correlation coefficients with the positive food preference score were higher for the Spanish population (r = 0.37; *p* < 0.001) than they were for the international students. However, similar results were obtained for the association between the total food preference score and global adherence to the MedDiet in the three groups: r = 0.39, *p* = 3.3 × 10^−27^ for participants from Valencia; r = 0.36, *p* = 1.5 × 10^−5^ for participants from other Spanish regions and r = 0.44, *p* = 0.005 for international students), adding external validity to these findings.

**Figure 3:**
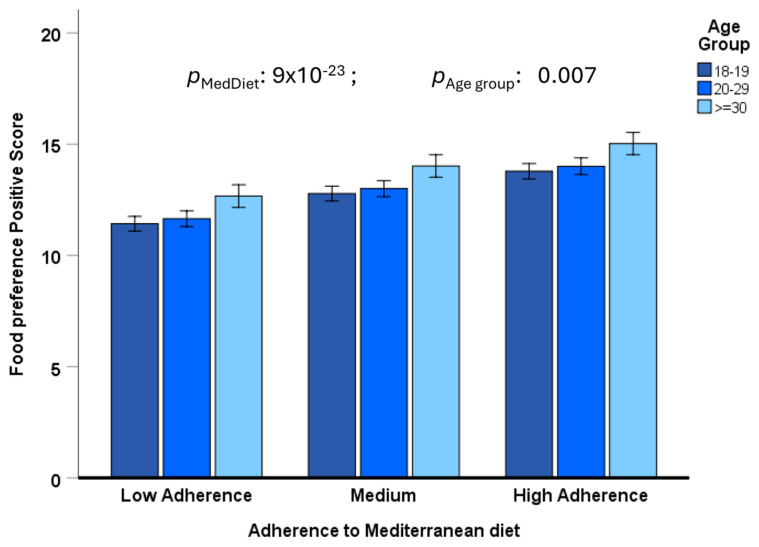
Differences by age groups (18–19 years, n = 668; 20–29 years, n = 173; and ≥30 years, n = 38) in the association between the positive food preference composite score and adherence to the Mediterranean diet (three levels: low, medium, and high) in the whole population. The bars are the adjusted means ± SE in the fully multivariate model (sex, geographical origin, BMI, smoking, drinking, physical activity, and sleep duration).

Despite the similar correlations between the food preference scores and adherence to the MedDiet among the three age groups, we were interested in investigating whether the positive and negative food preference scores differed by age group. Figure 3 shows the mean differences by age group in the food preference positive score in the three different groups of adherence to the MedDiet, after fully multivariate adjustment for sex, geographical origin, BMI, smoking, drinking, physical activity, and sleep (Model 3).

**Figure 4:**
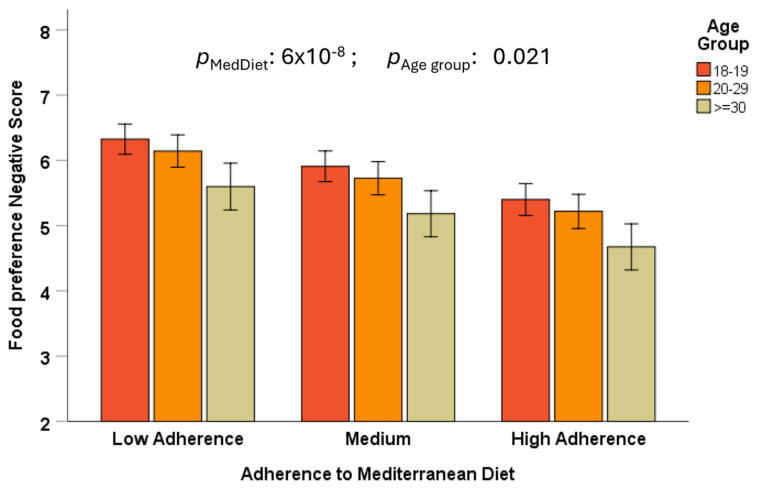
Differences by age groups (18–19 years, n = 668; 20–29 years, n = 173; and ≥30 years, n = 38) in the association between the negative food preference composite score and adherence to the Mediterranean diet (three levels: low, medium, and high) in the whole population. The bars are the adjusted means ± SE in the fully multivariate model (sex, geographical origin, BMI, smoking, drinking, physical activity, and sleep duration).

Despite a high association with the level of adherence to the MedDiet, we observed a statistically significant effect of age group (*p* = 0.007), in such a way that the positive food preference score was lower in the younger age group (18–19 years) in comparison with subjects aged ≥ 30 years, with a linear trend. This suggests lower adherence to the MedDiet in younger people. Similarly, we observed an inverse age group effect (*p* = 0.021 in Model 3) for the negative total food preference score (Figure 4). Preference for food with a negative score (red meat, sweet foods, and butter) was higher among the younger participants (aged 18–19), with an inverse trend.

### 3.4. Association Between Food Preference for Individual Food Items and Compliance with the Related Food Items in the 14-Item MedDiet Score

In addition to analyzing the association of each food or the composite food score with overall adherence to the MedDiet, we found it fascinating to investigate the association between the preference for each food and the compliance with the recommendation for its consumption on the MedDiet adherence scale 14-MEDAS. Table 5 shows the associations between the preference for the selected foods and their corresponding items on the Mediterranean diet scale.

We estimated the OR, indicating associations with greater adherence, and adjusted according to Model 3, including for sex, age, geographical origin, BMI, smoking, drinking, physical activity, and sleep duration.

We obtained strongly significant associations of adherence with all foods, except for butter. The OR indicates the increase in compliance with the food item, according to the 14-MEDAS scale, per unit of increase in the four-point Likert scale for the corresponding food preference. Highly relevant associations were obtained for fruits (OR: 2.9; *p* = 4.0 × 10^−20^), legumes (OR: 2.5; *p* = 5.0 × 10^−22^), nuts (OR: 2.6: *p* = 1 × 10^−19^), and fish (OR: 2.0; *p* = 1.0 × 10^−16^).

### 3.5. Taste Preferences and Associations with Adherence to the MedDiet

In addition to preferences for specific foods, we assessed the degree of liking for different tastes. Using a similar ascending Likert-type four-point scale, we measured the preference ratings for bitter, sweet, salty, and sour tastes. Sweet (2.54 ± 0.02) was the most preferred taste modality in the whole population, with salty (2.42 ± 0.02), bitter (0.90 ± 0.03), and sour (0.79 ± 0.03) following, in that order. Statistically significant differences were observed in the preferences for bitter and sweet tastes by sex (*p* < 0.05). Men exhibited a greater preference for bitter taste, while women favored sweet taste, albeit with minor variations. The preference for umami taste was not asked about, because it was not included in the initial questionnaire, as the original four basic tastes did not include umami. We did not detect statistically significant differences in mean preference by age groups (*p* > 0.05).

When we analyzed the association between taste preference for the different taste modalities and adherence to the MedDiet (Table 6) in the whole population, we observed highly statistically significant associations for bitter and sweet taste preferences. For bitter taste, higher preference was directly associated with higher adherence to the MedDiet (*p* = 0.004 in the fully adjusted Model 3).

On the other hand, this association was inverse for those who preferred sweet taste. We detected higher adherence to the MedDiet in subjects having the lowest preference for sweet taste (*p* < 0.001 in Model 3). We found no significant associations for salty taste. Model 3 found a small statistical significance (*p* = 0.027) for sour taste preference, as higher adherence was associated with a higher sour taste preference.

**Figure 5:**
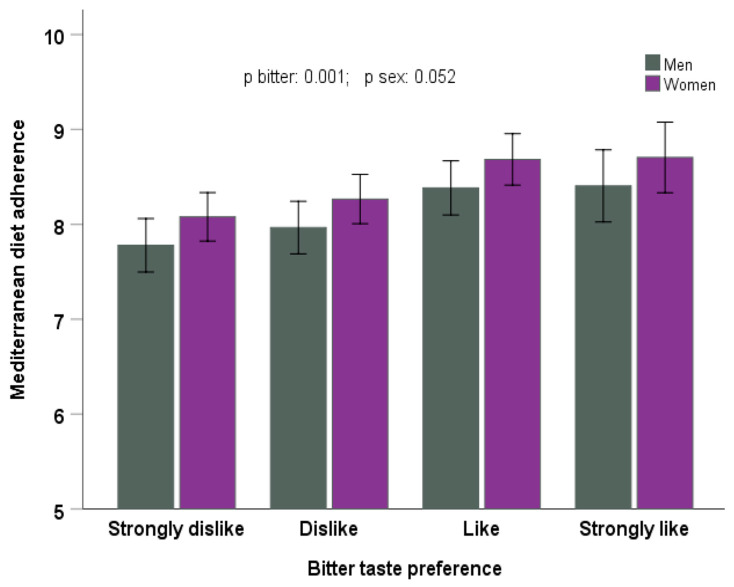
Adherence to the Mediterranean diet depending on bitter taste preference and sex (men, n = 264; women, n = 615), in the whole population. The bars are the adjusted means ± SE in the model adjusted for age, geographical origin, BMI, smoking, drinking, physical activity, and sleep duration.

In the analysis by sex (Figure 5), we observed that bitter taste preference was directly associated with higher adherence to the MedDiet (as continuous) in both men and women (*p* = 0.001 in the model adjusted for age, geographical origin, BMI, smoking, drinking, physical activity, and sleep duration).

**Figure 6:**
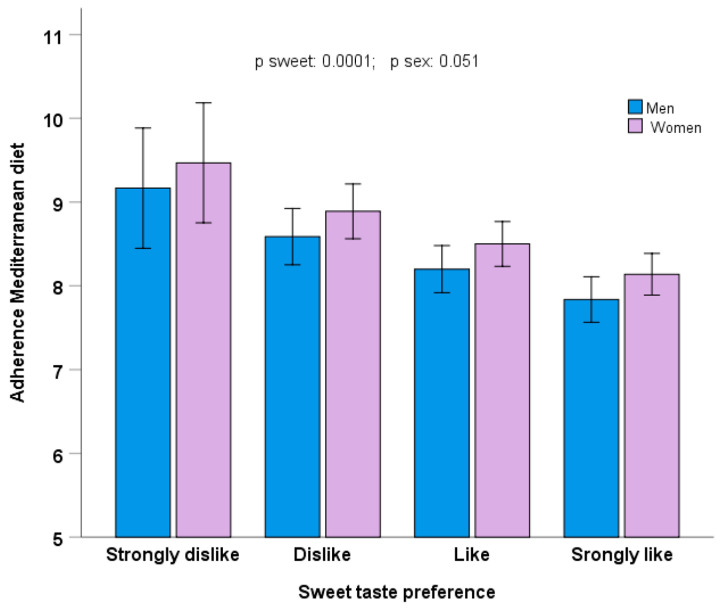
Adherence to the Mediterranean diet depending on sweet taste preference and sex (men, n = 264; women, n = 615) in the whole population. The bars are the adjusted means ± SE in the model adjusted for age, geographical origin, BMI, smoking, drinking, physical activity, and sleep duration.

Likewise, for sweet taste preference, we observed that higher preference for this taste was inversely associated with adherence to the MedDiet (as continuous) in both men and women (*p* = 0.0001) in the model adjusted for age, geographical origin, BMI, smoking, drinking, physical activity, and sleep duration (Figure 6).

Additional adjustment of the models for the four taste modalities in a joint model resulted in a statistically significant inverse association for sweet taste (*p* < 0.05), and independent direct associations (*p* < 0.05) for bitter taste. The joint adjustment eliminated the statistical significance for sour taste in the separate model.

### 3.6. Taste Perception and Association with Adherence to the MedDiet

Finally, we tested the taste perception of participants in the laboratory, using five prototypical tastants for the five taste modalities (bitter, sweet, salty, sour, and umami), as indicated in the Methods Section. We used five concentrations for each tastant (in addition to the zero concentration control with distilled water). Supplementary Figure S3 shows the ratings (means and SE) of perceived taste intensity in response to the different tastant concentrations (I–V, indicated in the Figure S3) for sweet, salty, sour, and umami tastes for each of the concentrations tested, scored on a scale from 0 to 5. Only these tastes are depicted in Figure S3, as they were tasted in liquid solution (bitter was tested on filter paper, and similar results were found). As the tastant concentration increased, the rating score for all of the tastants also increased. Therefore, for the associations with adherence to the MedDiet, we only used the data obtained after testing concentration V for the five tastants. In addition to the separate perception for the taste modalities, we calculated a composite total taste preference score for concentration V, as indicated in Methods. This composite score ranged from 0 to 25. Supplementary Figure S4 presents the frequency distribution for the composite score.

**Figure 7:**
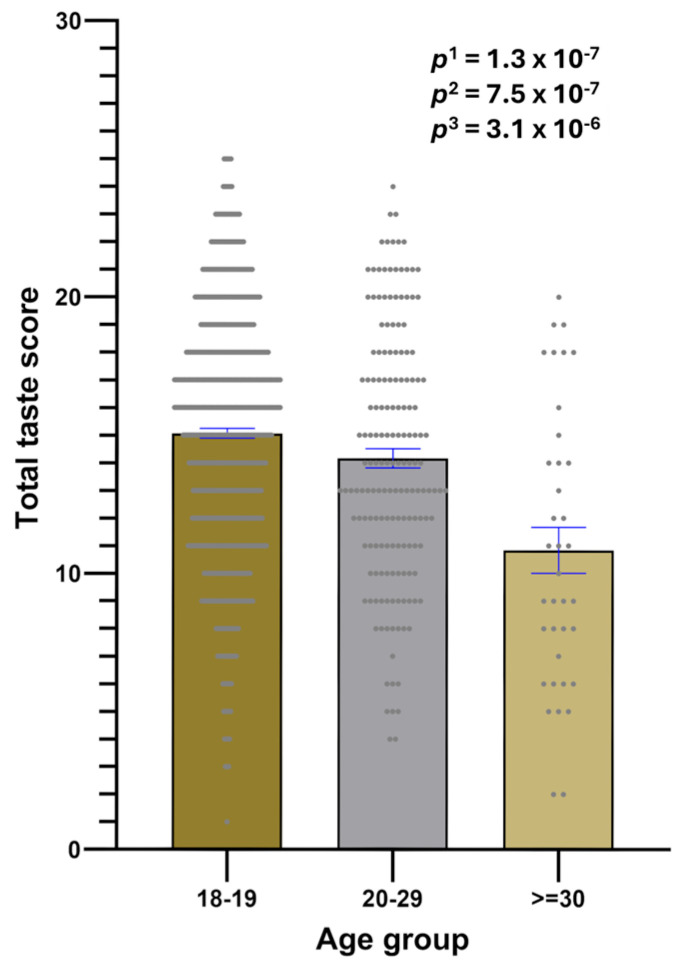
Associations between the total taste perception score for the five taste modalities at concentration V, and the age groups (18–19 years, n = 668; 20–29 years, n = 173; and ≥30 years, n = 38), in the whole population. The bars are the mean values ± SE for the total taste score. The means and dots for each group are depicted. *p*^1^: unadjusted model. *p*^2^: model adjusted for sex and geographical region. *p*^3^: model additionally adjusted for BMI, smoking, drinking, physical activity, and sleep duration.

The total taste score was slightly higher in women than in men, but this difference did not reach statistical significance (14.8 ± 0.18 vs. 14.3 ± 0.28; *p* = 0.094). However, we detected a statistically significant difference in total taste score perception across age groups. Figure 7 shows the mean differences in the total taste score by the three age groups; the mean decreased significantly in the oldest group (*p* = 3 × 10^−6^) in the model adjusted for sex, geographical origin, BMI, smoking, drinking, physical activity, and sleep duration.

Further, we analyzed (Table 7) the association between taste perception (concentration V for each taste modality) and adherence to the MedDiet (as continuous). We only detected a statistically significant inverse association of bitter taste perception with adherence to MedDiet (B = −0.076 ± 0.038; *p* = 0.049 in the model adjusted for age, sex, geographical origin, BMI, smoking, drinking, physical activity, and sleep duration). Despite the fact that sweet taste perception was positively associated with adherence, the results did not reach the statistical significance. Likewise, no statistically significant association was detected for the total taste perception score (*p* = 0.347 in Model 3).

### 3.7. Associations Between Food Liking, Taste Preferences, Taste Perception, and MedDiet Adherence

**Figure 8:**
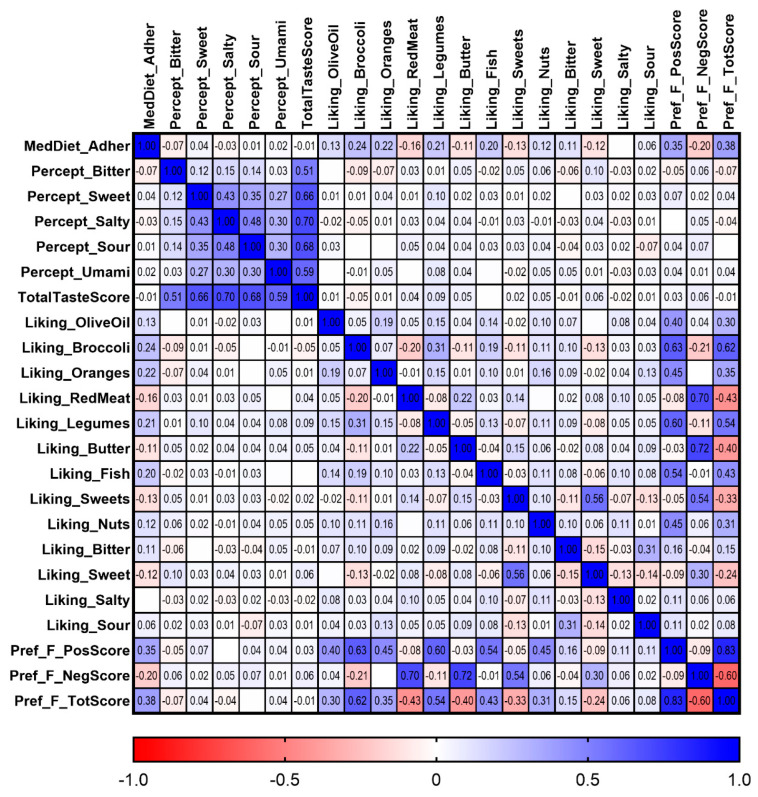
Heatmap of correlations between the following: adherence to Mediterranean diet; taste perception for bitter, sweet, salty, sour, and umami; taste preference for bitter, sweet, salty, and sour; selected food preferences; and composite scores for food preference and for taste perception, in whole population (n = 877).

Finally, we analyzed the associations between food liking, taste preferences, taste perception, and adherence to the MedDiet in the whole population. Figure 8 shows a heatmap of the correlations among all these variables. Spearman correlation coefficients were computed. As previously presented (Figure 1), we detected strong and highly significant correlation coefficients between the composite scores for food preferences (positive, negative, and total) and adherence to the MedDiet. However, the correlation coefficient between the total taste preference score and adherence to the MedDiet was small, and did not reach statistical significance (*p* > 0.05).

Interestingly, bitter taste perception was inversely correlated with adherence to the MedDiet (r = −0.07; *p* = 0.046), and significantly correlated with preference for some foods: r = −0.09; *p* = 0.004 for broccoli and r = −0.07; *p* = 0.047 for oranges, as well as significantly correlated with total food preference score (*p* = −0.07; *p* = 0.036). Moreover, bitter taste perception was inversely associated with liking for sweet taste (r = −0.11; *p* = 0.003), and borderline associated with liking for bitter taste (r = −0.06; *p* = 0.053). In terms of taste perception modalities, we observed strong statistically significant correlations between some of them (for example, between salty and sour perception: r = 0.48; *p* = 3.9 × 10^−52^).

Higher sweet perception was positively correlated with liking for legumes (r = 0.1; *p* = 0.004), and with the positive food preference score (r = 0.07; *p* = 0.042). A higher perception of sour taste was inversely correlated with preference for sour taste (r = −0.07; *p* = 0.042), as well as being directly associated with the negative food preference score (r = 0.07; *p* = 0.040). In the heatmap, we can observe other strong correlations among liking for specific foods and liking for specific tastes, as well as with food preference scores (positive, negative, and total). In general, correlation coefficients higher than 0.06 are statistically significant.

## 4. Discussion

This study, which was conducted on a predominantly young university population, presents novel and compelling findings regarding additional factors that enhance adherence to the MedDiet, which have largely been ignored in previous studies analyzing factors associated with level of compliance with the MedDiet [51,52,53,54,55,56,57,58,59,60,61,62,63,64,65,66,67,68,69,70].

To the best of our knowledge, this is the first study to examine the impacts of hedonic taste preferences and liking for specific foods on global adherence to the MedDiet using a validated scale [112]. MedDiet adherence was evaluated using the MEDAS score, which is a widely used tool [112]. In addition to the level of adherence using the global MedDiet score, we examined the association between liking for individual foods and compliance with the scale’s recommendations for these specific foods. The results of our investigation indicate that the MedDiet was more readily adhered to when participants liked particular foods, including olive oil, certain vegetables, certain fruits, legumes, nuts, and fish. The stronger the preference for these foods, the stronger the correlation with adherence to the overall dietary pattern, even after additional adjustment for socio-demographic and lifestyle factors (smoking, drinking, physical activity, and sleep duration). Furthermore, alongside individual analyses for each specific food, we developed composite taste preference scores that incorporated the additive preferences for each particular food. Therefore, we computed a “food preference positive score” that included foods positively associated with adherence to the MedDiet. After analyzing the association between adherence and this “food preference positive score”, we obtained a prominent statistically significant association of this score with adherence to the MedDiet (*p* < 1.4 × 10^−23^), that remained statistically significant for the multivariable adjusted model (*p* < 1.4 × 10^−23^). This robust correlation was independent of sex, age, geographical origin, BMI, smoking, drinking, physical activity, and sleep duration. We are unable to rule out the possibility of a bias effect from other unmeasured confounders. However, we can hypothesize a small impact of other potential confounders, given the strong association. In addition, our results reveal that higher preference for foods such as red meats, sweet foods, and butter was associated with lower adherence to this dietary pattern. Then, we computed the “food preference negative score” and found a strong inverse association between this score and adherence to the dietary pattern. Moreover, we reversed the preferences of the negative score and added the rating of the corresponding food to the “food preference positive score”, creating the so-called “total preference food score”. This score was still more significantly associated with global adherence to the MedDiet pattern, revealing that both increased preference for healthy foods and reduced preference for less healthy foods contributed to increasing adherence to the MedDiet in this population. This strong association remained statistically significant even after adjusting for the primary potential confounders that were previously mentioned (sex, age, geographical origin, BMI, smoking, drinking, physical activity, and sleep duration). As far as we know, this is the first time that these combined food preference scores have been analyzed in association with the MedDiet pattern. Previous studies analyzing food preferences and adherence to the MedDiet have been extremely restricted in scope, and have concentrated on a limited number of specific foods or specific taste modalities [71,72,73,74,75,76,77]. Our findings indicate that food preferences, both in terms of individual food items, and, more importantly, as combined food scores, play a crucial role in determining food consumption, and should be considered in both current and future studies examining the factors linked to adherence to the MedDiet. This is a significant finding for precision nutrition [25,26,27,28], due to the fact that we have identified a relevant factor (food preference) that is significantly correlated with higher or reduced MedDiet adherence, and exhibits inter-individual variation. This finding may have implications for the development of dietary interventions for young adults, with the goal of enhancing adherence to the MedDiet by focusing on individual characteristics, rather than a one-size-fits-all approach [25,26,27,28,29,30,31,32].

Although our results are pioneering in the research of factors associated with adherence to the MedDiet, there have been several initial studies analyzing the influence of food preferences/liking on consumption, as well as the association of these factors with other healthy eating patterns or phenotypes related to obesity [82,83,89,90,94,99,102,103,104,105,106,107,114,118,119]. In general, a strong association between liking for certain foods or food groups and their intake has been reported [82,90,94,99,102,103,104,105,106,107,114,118,119]. However, these studies did not specifically focus on MedDiet adherence. Among food liking studies associated with dietary patterns, we would like to mention the study carried out by Wanich et al. [119] on Australian young adults. This study has several points in common with the present research. The study took place among university students involved the health and nutrition field, with a mean age of 21.9 years, and a female sample that was more than three times the size of the male sample. In addition, the prevalence of obesity was very low. Participants completed a food liking questionnaire (using a hedonic scale) and a food frequency questionnaire to assess food intake. Rather than concentrating on MedDiet adherence, the authors calculated diet quality based on compliance with the Dietary Guideline Index (DGI) for Australia [119]. Moreover, the authors calculated several liking scores by grouping specific food items together. Liking scores were obtained for the following food items: grains, vegetables, fruits, dairy, animal-based proteins, fat and oil, sweet foods, salty foods, and alcohol. Strong direct associations were found between liking for healthy foods and compliance with the DGI recommendations. Likewise, inverse scores were found for liking for the unhealthy food groups and compliance with the DGI recommendations. This study [119] and our study add to the existing literature more evidence that food liking is a relevant driver of food intake and adherence to healthy dietary patterns, in our case, the MedDiet pattern.

In addition to food preferences, we analyzed taste preferences (for bitter, sweet, sour, and salty tastes), and found a strong inverse association between a higher preference for sweet taste and global adherence to the MedDiet, as well as a strong association with preference for sweet foods. Likewise, we detected another strong independent association between higher preference for bitter taste and higher adherence to the MedDiet, as well as with a higher preference for bitter foods, in our study. While they have not focused on patterns of adherence to the MedDiet, multiple prior studies have examined the association between a greater preference for sweet taste, the consumption of less healthy foods (pastries, ice cream, sugar-sweetened beverages, etc.), and a trend towards a less healthful dietary pattern [74,83,89,90,99,101,102,105,107,120,121,122]. Likewise, some prior research has identified associations between liking for bitter taste, intake of healthier foods (olive oil, broccoli, artichokes, spinaches, nuts, legumes), and dietary patterns [75,83,89,95,102,104,122,123]. Our study supports prior findings, and further highlights the negative (for sweet taste preference) and positive (for bitter taste preference) associations between these taste preferences and increased adherence to the MedDiet. Again, these findings may be very useful for precision nutrition and precision health [25,26,27,28,29,30,31,32].

Once the strong association between food and taste preferences and greater adherence to the MedDiet is known, it is necessary to investigate the factors that may explain this association. This research is not easy, as it is hypothesized that there may be multiple genetic and environmental factors involved. Our group conducted a genome-wide association study (GWAS) in an elderly Mediterranean population [74] to investigate the potential genetic variants associated with preference for sweet taste. Our findings showed that a polymorphism in the PTPRN2 (Protein Tyrosine Phosphatase Receptor Type N2) gene (minor allele) was significantly associated with a lower preference for sweet taste at the GWAS level. Other studies that have analyzed the genetics of sweet taste preferences have shown mixed results [99,124,125,126]. Moreover, in the UK Biobank, including more than 100,000 participants, liking for over 139 specific foods was investigated at the GWAS level [126]. This study identified 1401 significant food liking associations. However, this study and other similar studies are preliminary, and more research is needed on the genetics of food and taste preferences in different populations [127], based on genetic screening of these variants [25,26,27,28,29,30,31], for application in precision nutrition.

In addition to the genetics of food preferences and taste preferences, another one of the most researched factors related to these preferences and food consumption is the ability to perceive the basic taste modalities (bitter, sweet, salty, sour, and umami) [72,91,97,98,99,100,101,104,106,107,128,129,130,131,132,133,134,135]. It is known that there is great individual heterogeneity in taste perception. Thus, the same concentration of a prototypical tastant is perceived as very strong by some people, while others are unable to perceive anything. Genetic factors are strongly associated with bitter taste perception, mainly the polymorphism in the TAS2R38 gene [72]. For the other taste modalities, although some candidate genes have been identified (TAS1R1/2/3 genes for umami and sweet taste perception, among others), the consistency among different studies is low [72,91,97,98,99,101,103,104,105,106,107,128,129,130,131,132,133]. In the present study, we were unable to analyze genetic factors, but we did conduct objective laboratory tests for the perception of the five tastes. These tests are very laborious, and, therefore, have not been included in most epidemiological studies with large samples. This may contribute to the results not being consistent across different publications, since the sample size is usually small. Conversely, our study, with more than 850 participants, has a large sample size for this type of research, and therefore, our statistical power is greater. Additionally, we used previously validated taste perception tests that have been used in other previous studies we conducted in our laboratory [97,98]. In comparison with food preferences, we did not detect highly significant associations between taste perception and adherence to the MedDiet. Only for bitter taste perception did we find a statistically significant inverse association with adherence to the MedDiet. This association was small, but consistent with other publications and with the inverse association with liking for bitter foods. Likewise, in our study, we observed a direct association between bitter taste perception and sweet taste liking, as well as a significant inverse association between bitter food perception and the total taste preference score.

In general, several previous studies have reported an inverse association between food perception and taste preference or food preferences [98,99,101,102,103,104,105], and our research confirms some of the previous findings. However, further research is necessary to gain a deeper understanding of the role of other factors in explaining food preferences, considering the limited relevance of taste perception. There are several studies focusing on the microbiota [134,135], but additional factors should be investigated. More importantly, further research is required to gain a more comprehensive understanding of the cultural factors that influence food preferences, and how they can be modified to increase adherence to the MedDiet by shifting food preferences toward healthier options.

Our study has strengths and limitations. The main strengths are the novelty in the study’s research methods and relevant findings, and the relatively high sample size for a study testing taste perception for the five taste modalities with several molar concentrations of prototypical tastants, as well as the integration of measures of taste perception, taste preferences, food preferences, and adherence to the MedDiet at the same time. Moreover, we assessed other relevant socio-demographic and lifestyle factors (smoking, drinking, physical activity, and sleep duration), and tested their influence as potential confounders, thus minimizing bias for the main confounding factors. In addition, despite the fact that the great majority of the population was young, we analyzed three age groups and compared the associations, obtaining similar findings. Moreover, in our research, we included participants from the Valencia region, participants from other regions of Spain, and international students. Although the sample size for international students was low, we analyzed the main associations, taking into account geographical origin, and found similar associations in international students, increasing the external validity of our findings.

Our study has several limitations. One limitation is that we carried out a cross-sectional study, and more follow-up longitudinal studies are needed to better understand how the associations between food preferences, taste preference, taste perception, and food intake vary, as well as the related factors. One additional limitation of this study is the inclusion of only medical students. Due to their high cultural and socio-economic status, as well as their superior health training compared to other population groups, we cannot determine whether the results would hold true for other populations. However, on the other hand, analyzing homogeneous individuals in terms of socio-cultural variables can be an advantage, as it allows us to better study the factors that determine inter-individual differences without the confusion caused by unexamined social variables related to educational level. Similarly, the increased proficiency of the analyzed participants may increase the validity of the results from questionnaires and taste perception tests, thereby minimizing the impact of bias. Another limitation we would have liked to overcome is the absence of genetic and epigenetic tests that could have provided valuable additional information about the influence of these determinants and the possible application of genetic testing for the main genetic variants in precision health.

## 5. Conclusions

In this investigation, we identified a robust association of preference for specific foods and tastes with adherence to the MedDiet. This association is not influenced by the main confounding factors that were investigated, and may have significant implications for personalized or precision nutrition. Since an increase in adherence to the MedDiet has been associated with a lower incidence of chronic diseases, it is essential to understand the inter-individual factors associated with greater or lesser adherence, in order to intervene with these factors. Surprisingly, most of the major studies on the MedDiet have not incorporated the measurement of food and taste preferences. Consequently, the results of our study suggest that it would be beneficial to incorporate these measurements into future studies, in order to gain a more comprehensive understanding of the relationship between these variables in different populations. Furthermore, it is crucial to conduct a more comprehensive examination of the factors that influence dietary preferences and their dynamic evolution, in order to develop novel precision health intervention programs that are more specifically tailored to the unique characteristics of each individual.

## Figures and Tables

**Table 1:** Demographic, clinical, and lifestyle characteristics of the study population, according to sex.

	Total	Men	Women	*p* ^1^
	(*n* = 879)	(*n* = 264)	(*n* = 615)	
Age (years)	20.46 ± 0.16	21.31 ± 0.36	20.10 ± 0.16	<0.001
Weight (kg)	62.06 ± 0.38	73.37 ± 0.56	57.21 ± 0.34	<0.001
Height (m)	1.69 ± 0.00	1.78 ± 0.00	1.65 ± 0.00	<0.001
Body mass index (kg/m^2^)	21.67 ± 0.10	23.05 ± 0.16	21.07 ± 0.11	<0.001
Systolic blood pressure (mm Hg)	110.62 ± 0.39	119.92 ± 0.67	106.64 ± 0.39	<0.001
Diastolic blood pressure (mm Hg)	65.67 ± 0.27	67.01 ± 0.52	65.09 ± 0.31	0.002
Heart rate (beats/min)	74.40 ± 0.38	71.11 ± 0.71	75.79 ± 0.44	<0.001
Sleep duration (hours)	6.53 ± 0.03	6.53 ± 0.05	6.53 ± 0.03	0.948
MedDiet adherence ^2^	8.54 ± 0.07	8.51 ± 0.12	8.55 ± 0.08	0.746
Age groups				<0.001
*18 to 19 years*	76.0%	67.4%	79.7%	
*20 to 29 years*	19.7%	25.4%	17.2%	
*≥30 years*	4.3%	7.2%	3.1%	
Body mass index (BMI) (kg/m^2^)				<0.001
*BMI < 25*	89.4%	78.8%	94.0%	
*BMI 25–29.9*	9.4%	20.1%	4.9%	
*BMI ≥ 30*	1.1%	1.1%	1.1%	
Current smoker	6.3%	9.1%	5.1%	0.023
Former smoker	3.7%	3.8%	3.6%	0.887
Alcohol consumption (non-drinker)	14.4%	11.4%	15.8%	0.088
Walk more than 20 min/day	88.5%	92.0%	87.1%	0.037
Practice physical exercise	70.5%	78.8%	67.0%	<0.001
Geographical origin				0.940
*Spain (Valencia)*	79.7%	79.5%	79.8%	
*Spain (other regions)*	15.7%	15.5%	15.8%	
*International*	4.6%	4.9%	4.4%	

Values are mean ± SE for continuous variables or (%) for categorical variables. ^1^ *p*-value for comparisons (means or %) between men and women. ^2^ Mediterranean diet (MedDiet) adherence, measured with 14-item scale.

**Table 2:** Adherence to each of the 14 items of the Mediterranean diet (MedDiet): adherence score for the whole population and by sex.

Food Items	1 Point ^1^	Total	Men	Women	*p* ^2^
1. Do you use olive oil as your main culinary fat?	Yes	95.1%	93.2%	95.9%	0.081
2. How much olive oil (tablespoons) do you consume in a given day?	≥4	68.9%	76.8%	65.5%	<0.001
3. How many vegetable servings do you consume per day?	≥2	65.9%	58.6%	69.1%	0.003
4. How many fruit units (including natural fruit juices) do you consume per day?	≥3	51.5%	54.0%	50.5%	0.341
5. How many servings of red meat, hamburger, or meat products do you consume per week?	<1	68.3%	54.8%	74.1%	<0.001
6. How many servings of butter, margarine, or cream do you consume per week?	<1	82.1%	83.3%	81.6%	0.554
7. How many sweet/carbonated beverages do you drink per week?	<1	75.1%	71.5%	76.7%	0.101
8. How much wine (glasses) do you drink per week?	≥7	1.9%	2.3%	1.8%	0.630
9. How many servings of legumes do you consume per week?	≥3	38.4%	43.3%	36.3%	0.050
10. How many servings of fish or shellfish do you consume per week?	≥3	45.5%	43.0%	46.6%	0.325
11. How many times per week do you consume commercial sweets or pastries, such as cakes, cookies, biscuits, or custard?	<3	56.4%	58.6%	55.5%	0.409
12. How many servings of nuts do you consume per week?	≥3	46.8%	54.0%	43.6%	0.005
13. Do you preferentially consume chicken, turkey, or rabbit meat instead of veal, pork, hamburger, or sausage?	Yes	76.9%	73.0%	78.5%	0.077
14. How many times per week do you consume vegetables, pasta, rice, or other dishes seasoned with sofrito?	≥2	81.0%	84.4%	79.5%	0.088
Total score (high adherence) in points	≥9	51.1%	51.8%	49.4%	0.521

Values are percentages of compliance with criteria of adherence to MedDiet (see Table S1 for more details). ^1^ criteria for 1 point are indicated. ^2^ *p*-values for differences by sex (men, n = 264; and women, n = 615).

**Table 3:** Correlations between food preferences (liking) and adherence to the Mediterranean diet (MedDiet) in the whole population.

	Adh MedDiet		
Food Preferences	rho ^1^	95% Confidence Interval	*p* ^2^
Whole milk	−0.073	(−0.141, −0.005)	0.030
Skimmed milk	0.025	(−0.043, 0.093)	0.462
Whole yogurt	−0.023	(−0.091, 0.045)	0.495
Skimmed yogurt	0.042	(−0.027, 0.110)	0.220
Eggs	0.028	(−0.041, 0.096)	0.417
Red meats	−0.164	(−0.229, −0.097)	<0.0001
Poultry	0.003	(−0.066, 0.071)	0.940
Ham, sausages	−0.105	(−0.172, −0.037)	0.002
White fish	0.203	(0.137, 0.267)	<0.0001
Blue fish	0.211	(0.145, 0.275)	<0.0001
Seafood	0.027	(−0.041, 0.095)	0.424
Cured cheese	0.068	(0.001, 0.135)	0.046
Fresh cheese	0.087	(0.019, 0.154)	0.010
Bread	0.021	(−0.047, 0.089)	0.529
Pasta	−0.102	(−0.169, −0.034)	0.003
Legumes	0.211	(0.144, 0.275)	<0.0001
French beans	0.244	(0.179, 0.307)	<0.0001
Broccoli, cauliflower	0.244	(0.179, 0.307)	<0.0001
Artichokes, spinach	0.220	(0.154, 0.284)	<0.0001
Oranges, mandarins	0.218	(0.152, 0.282)	<0.0001
Lemon	0.109	(0.041, 0.176)	0.001
Other fruits	0.170	(0.102, 0.235)	<0.0001
Nuts	0.122	(0.054, 0.189)	<0.001
Pastries	−0.133	(−0.199, −0.065)	<0.0001
Chocolate	−0.071	(−0.139, −0.003)	0.035
Sugar	−0.144	(−0.210, −0.076)	<0.0001
Breakfast cereals	0.003	(−0.065, 0.071)	0.933
Olive oil	0.134	(0.066, 0.200)	<0.0001
Sunflower oil	−0.142	(−0.209, −0.075)	<0.0001
Other oils	−0.056	(−0.124, 0.012)	0.098
Butter	−0.112	(−0.179, −0.045)	0.001
Margarine	−0.112	(−0.179, −0.044)	0.001

Food preferences were assessed using a 4-point Likert scale, and adherence (Adh) to the MedDiet using a 14-item scale. ^1^ Spearman correlation coefficient and 95% confidence interval. ^2^ *p*-value for Spearman correlation (n = 877).

**Table 4:** Associations between the scores for food preference and adherence to the Mediterranean diet (MedDiet) in the whole population.

Food Scores	Adherence to Mediterranean Diet			*p* ^1^	*p* ^2^	*p* ^3^
	Low (1–7)	Medium (8–9)	High (10–14)			
Positive score	11.22 ± 0.17	12.48 ± 0.14	13.52 ± 0.15	1.223 × 10^−23^	4.965 × 10^−24^	1.373 × 10^−23^
Negative score	6.21 ± 0.12	5.77 ± 0.09	5.27 ± 0.11	2.954 × 10^−09^	1.871 × 10^−08^	1.986 × 10^−08^
Total score	14.01 ± 0.22	15.71 ± 0.16	17.26 ± 0.19	1.545 × 10^−29^	1.781 × 10^−29^	4.556 × 10^−29^

Values are mean ± SE for corresponding food score. Food scores were computed as Likert scores, adding values for selected foods. For positive score, positively associated foods were selected. For negative score, foods with negative correlations were selected. Adherence to MedDiet was expressed as three levels, depending on 14-item scale: low (n = 252); medium (n = 342); high (n = 283). ^1^ *p*-values in unadjusted model. ^2^ *p*-values in model adjusted for sex, age, and geographical origin. ^3^ *p*-values in additionally adjusted model for BMI, smoking, drinking, physical activity, and sleep duration.

**Table 5:** Associations between food preference and compliance with the corresponding item on the Mediterranean diet (MedDiet) scale in the whole population.

Item on MedDiet Scale	Food Preference	OR	95% (CI)	*p* ^1^
2. Olive oil servings	Olive oil	1.58	(1.25–1.49)	1.0 × 10^−4^
3. Vegetable intake	Broccoli	1.79	(1.54–2.07)	9.0 × 10^−15^
4. Fruit units	Oranges	2.93	(2.33–3.68)	4.0 × 10^−20^
5. Read meat servings	Read meat	0.71	(0.60–0.85)	1.0 × 10^−4^
6. Butter	Butter	1.15	(0.96–1.39)	0.122
9. Legumes	Legumes	2.51	(2.08–3.01)	5.0 × 10^−22^
10. Fish	White fish	2.03	(1.72–0.69)	1.0 × 10^−16^
11. Pastries	Pastries	0.58	(0.47–0.69)	2.0 × 10^−8^
12. Nuts	Nuts	2.57	(2.09–3.15)	2.0 × 10^−19^

Values are OR and 95% confidence interval (CI) for compliance with Mediterranean diet item per 1-unit increase in category of food preference (from 0 to 3). ^1^ *p*-values obtained in fully adjusted models, including sex, age, geographical origin, BMI, smoking, drinking, physical activity, and sleep duration.

**Table 6:** Adherence to the Mediterranean diet (MedDiet) depending on the taste preference modality in the whole population.

	Taste Preference Level						
Taste Modalities	0	1	2	3	*p* ^1^	*p* ^2^	*p* ^3^
Bitter	8.35 ± 0.10	8.48 ± 0.11	8.93 ± 0.15	8.91 ± 0.36	0.027	<0.001	0.004
Sweet	9.50 ± 0.96	9.05 ± 0.27	8.76 ± 0.13	8.37 ± 0.08	0.078	<0.001	<0.001
Salty	8.57 ± 0.62	8.68 ± 0.25	8.45 ± 0.11	8.56 ± 0.09	0.877	0.942	0.949
Sour	8.45 ± 0.09	8.47 ± 0.11	8.81 ± 0.18	8.90 ± 0.35	0.089	0.031	0.027

Values are mean ± SE for adherence to MedDiet. Taste preferences for each modality are expressed on Likert scale from zero (strongly dislike) to three (strongly like). ^1^ *p*-values in unadjusted model. ^2^ *p*-values adjusted for sex, age, and geographical origin. ^3^ *p*-values additionally adjusted for BMI, smoking, drinking, physical activity, and sleep duration.

**Table 7:** Associations between taste perception modalities and adherence to the Mediterranean diet (MedDiet) in the whole population.

Taste Modalities (Concentration V)	Adherence to the MedDiet	*p* ^1^	*p* ^2^	*p* ^3^
	β ± SE			
Bitter (PROP)	−0.076 ± 0.038	0.044	0.046	0.049
Sweet (sucrose)	0.044 ± 0.049	0.366	0.264	0.303
Salty (NaCl)	−0.076 ± 0.052	0.143	0.196	0.132
Sour (citric acid)	0.001 ± 0.049	0.986	0.867	0.859
Umami (glutamate)	0.002 ± 0.044	0.962	0.986	0.970
Total taste score	−0.014 ± 0.015	0.338	0.432	0.347

Values are regression coefficients (Beta) ± SE of MedDiet score for 1-unit increase in level of perception for each taste modality in whole population (n = 879). Prototypical tastants for each taste modality at concentration V were rated (5.6 mM in PROP; 34 mM in Sucrose; 200 mM in NaCl; 400 mM in Citric Acid; 200 mM in Glutamate). Total taste score is composite of five ratings at concentration V. *p*^1^: *p*-values in unadjusted model. *p*^2^: *p*-values in model adjusted for sex, age, and geographical origin. *p*^3^: additionally adjusted for BMI, smoking, drinking, physical activity, and sleep duration.

## Data Availability

Neither the participants’ consent forms nor the ethics approval included permission for open access. However, we follow a controlled data-sharing collaboration model, and data for collaborations will be available upon request, pending application and approval. Investigators who are interested in this study can contact the corresponding author.

## Supplementary Materials

The following supporting information can be downloaded at https://www.mdpi.com/article/10.3390/nu17030600/s1: Table S1: Quantitative 14-item questionnaire for adherence to MedDiet; Table S2: Differences in food preference for selected foods between men and women; Figure S1: Frequency distribution of composite food preference scores; Figure S2: Correlation between selected food preference scores and adherence to MedDiet, stratified by geographical origin; Figure S3: Ratings of perceived taste intensity in responses to five concentrations; Figure S4: Frequency distribution of composite total taste score.
