# Supplementary material for: From Liking to Following: The Role of Food Preferences, Taste Perception, and Lifestyle Factors in Adherence to the Mediterranean Diet Among Young Individuals
Source: Nutrients. 2025 Feb 6;17(3):600. doi: 10.3390/nu17030600 (PMC11820210; doi:10.3390/nu17030600)

ONLINE SUPPORTING MATERIAL

From liking to following: The role of food preferences, taste perception, and lifestyle factors on adherence to the Mediterranean diet among young individuals

José V. Sorlí, Edurne de la Cámara, José I. González, Olga Portolés, Ignacio M. Giménez-Alba, Rebeca Fernández-Carrión, Oscar Coltell, Inmaculada Gonzalez-Monje, Carmen Saiz, Eva C. Pascual, Laura V. Villamil, Dolores Corella, Eva. M. Asensio, Carolina Ortega-Azorín

Supplemental Table S1 2
Supplemental Table S2 3
Supplemental Figure S1 4
Supplemental Figure S2 5
Supplemental Figure S3 5
Supplemental Figure S4 6

Supplemental Table S1. Quantitative 14-item questionnaire for Adherence to the Mediterranean diet

Food items and frequency of consumption	Criteria for 1 point[1]
1. Do you use olive oil as main culinary fat?	Yes
2. How much olive oil (tablespoons) do you consume in a given day (including oil used for frying, salads, out of house meals, etc.)?	≥4
3. How many vegetable servings do you consume per day? (1 serving = 200g [consider side dish as half serving]) (≥1 portion raw or as salad)	≥2
4. How many fruit units (including natural fruit juices) do you consume per day?	≥3
5. How many servings of red meat, hamburger or meat products (ham, sausage, etc.) do you consume per week? (1 serving = 100–150g)	<1
6. How many servings of butter, margarine, or cream do you consume per week? (1 serving = 12g)	<1
7. How many sweet/carbonated beverages (soft drinks, cola, bitter, juices without added sugars) do you drink per week?	<1
8. How much wine (glasses) do you drink per week?	≥7
9. How many servings of legumes do you consume per week? (1 serving = 150g)	≥3
10. How many servings of fish or shellfish do you consume per week? (1 serving = 100-150g of fish or 4-5 units or 200g of shellfish)	≥3
11. How many times per week do you consume commercial sweets or pastries (not homemade), such as cakes, cookies, biscuits, or custard?	<3
12. How many servings of nuts (including peanuts) do you consume per week? (1 serving = 30g)	≥3
13. Do you preferentially consume chicken, turkey, or rabbit meat instead of veal, pork, hamburger, or sausage?	Yes
14. How many times per week do you consume vegetables, pasta, rice, or other dishes seasoned with sofrito (sauce made with tomato and onion, leek, or garlic and simmered with olive oil)?	≥2

[1] '0' points if these criteria are not met.

Supplemental Table S2. Differences in food preference for selected foods between men and women.

Foods by sex	Food preference*				*p***
	0	1	2	3	
Legumes					0.309
Men	3.0%	29.7%	41.4%	25.9%	
Women	9.1%	25.8%	37.7%	27.4%	
Green beans					0.162
Men	14.1%	36.9%	33.5%	15.6%	
Women	18.6%	26.3%	30.7%	24.5%	
Broccoli, cauliflower, cabbage, etc.					0.024
Men	36.9%	32.3%	19.4%	11.4%	
Women	37.6%	19.9%	23.5%	19.0%	
Artichokes, spinach, chard					0.029
Men	17.5%	31.2%	29.7%	21.7%	
Women	17.9%	22.7%	28.1%	31.3%	
Oranges, tangerines					0.977
Men	2.3%	8.0%	27.8%	62.0%	
Women	4.2%	6.9%	24.3%	64.6%	
Other fresh fruits					0.225
Men	2.7%	8.0%	38.4%	51.0%	
Women	2.9%	8.2%	30.4%	58.5%	
Olive oil					0.746
Men	0.4%	6.8%	28.9%	63.9%	
Women	0.3%	5.4%	30.5%	63.8%	
Sunflower oil					0.002
Men	25.1%	43.0%	26.6%	5.3%	
Women	36.3%	39.4%	20.0%	4.3%	
Butter					0.416
Men	27.8%	33.5%	28.5%	10.3%	
Women	32.6%	30.8%	25.1%	11.4%	
Nuts					0.620
Men	3.0%	12.2%	32.7%	52.1%	
Women	3.4%	13.4%	32.1%	51.1%	
Pastries, ice cream					0.004
Men	3.8%	12.2%	32.7%	51.3%	
Women	3.3%	9.0%	23.5%	64.3%	
Ham, sausages					0.008
Men	4.9%	15.6%	27.0%	52.5%	
Women	9.1%	17.3%	29.9%	43.7%	
Red meats					<0.001
Men	3.8%	9.1%	25.9%	61.2%	
Women	9.8%	20.2%	31.5%	38.5%	
Blue fish					0.676
Men	7.2%	19.4%	36.1%	37.3%	
Women	9.0%	19.1%	34.4%	37.5%	
White fish					0.242
Men	6.5%	21.3%	39.9%	32.3%	
Women	7.2%	18.9%	34.6%	39.3%	

*: Preference rating from zero (strongly dislike) to three (strongly like). **: *P*-values for lineal trend differences by sex (Men, n=264; and Women, n=615).

Supplemental Figure S1. Frequency distribution of the composite food preference scores: positive score (A); negative score (B); and total score (C); in the whole population (n=879).

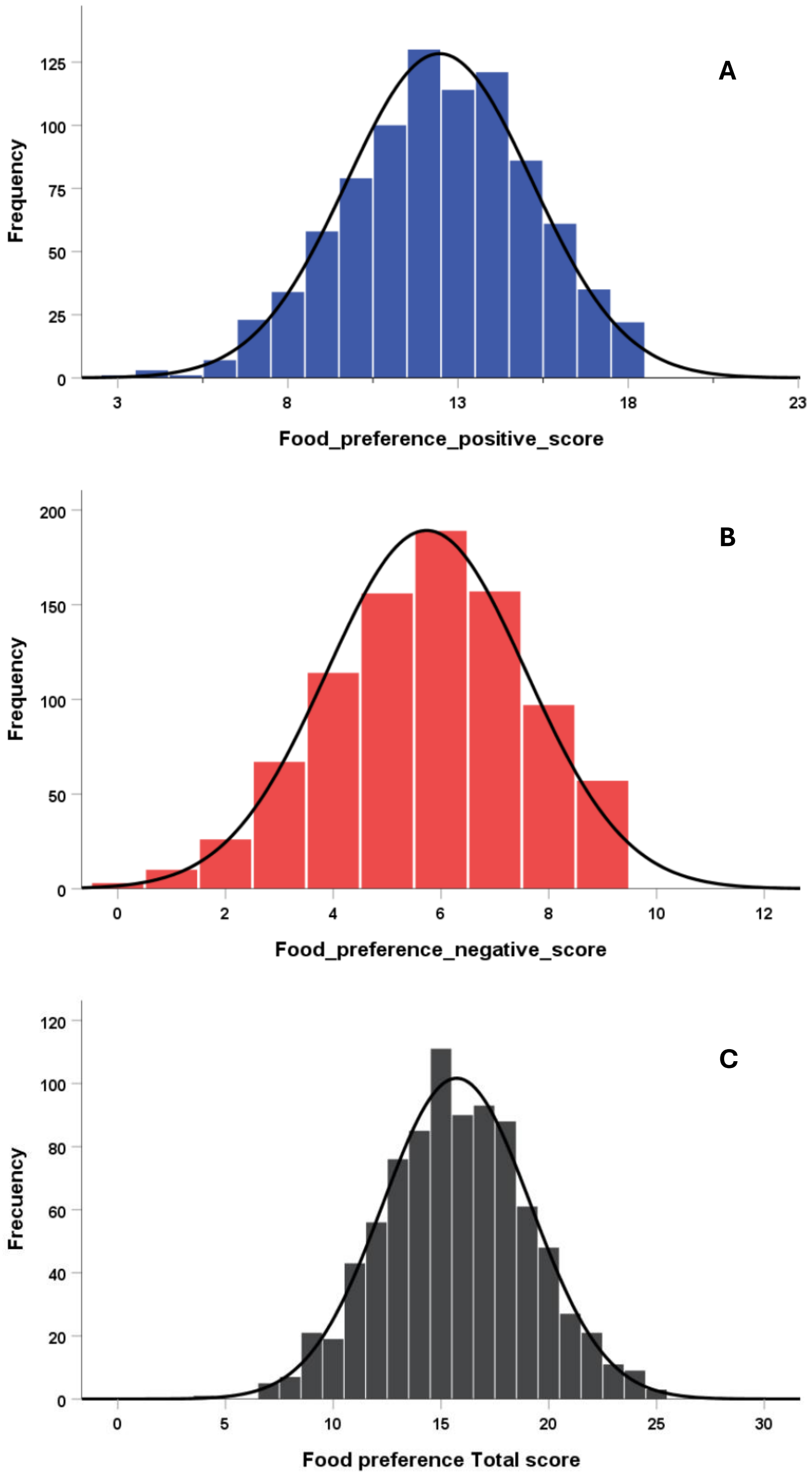

Supplemental Figure S2. Heatmaps of the correlation between selected food preference scores and adherence to the Mediterranean diet stratified by geographical origin (A: Valencia Region, n=701; B: other regions, n=138; C: international, n=40). Values are Pearson correlation coefficients.

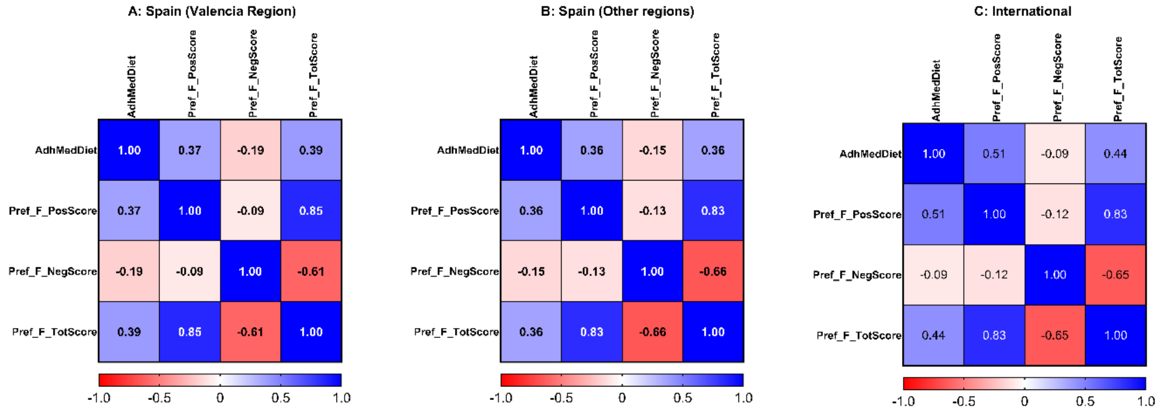

Supplemental Figure S3. Ratings of perceived taste intensity in response to five concentrations (I, II, III; IV and V) and distilled water as control of tastants for sweet, salty, sour, and umami in the whole population (n=879). Values are means ± SE.

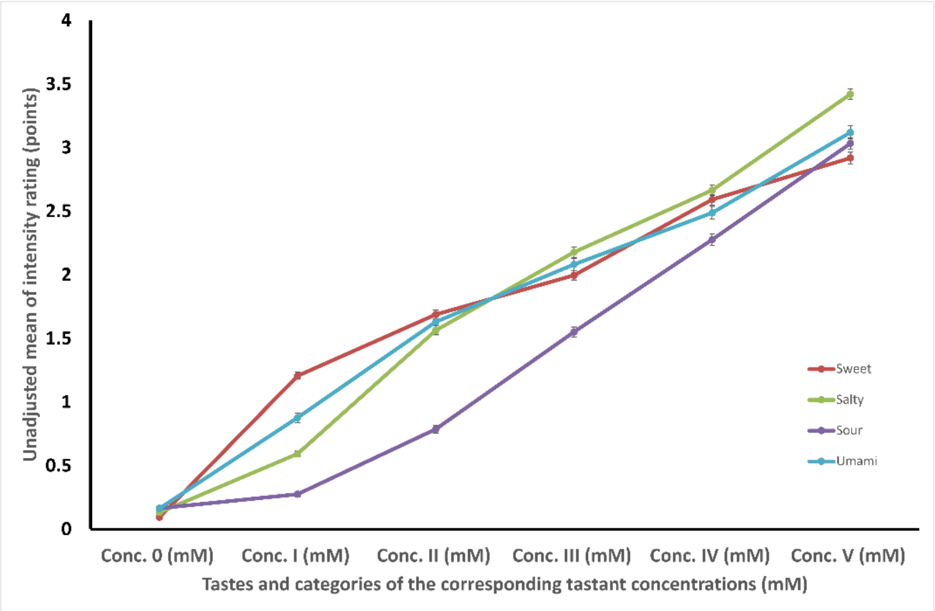

Taste	Tastant	Conc. 0 (mM)	Conc. I (mM)	Conc. II (mM)	Conc. III (mM)	Conc. IV (mM)	Conc. V (mM)
**Sweet**	Sucrose	0	1	5	10	17	34
**Salty**	NaCl	0	25	50	75	100	200
**Sour**	Citric acid	0	100	150	200	300	400
**Umami**	Glutamate	0	25	50	75	100	200

Supplemental Figure S4. Frequency distribution of the composite total taste score (sum of scores from the five taste modalities at concentration V for bitter, sweet, salty, sour and umami) in the whole population (n=879).

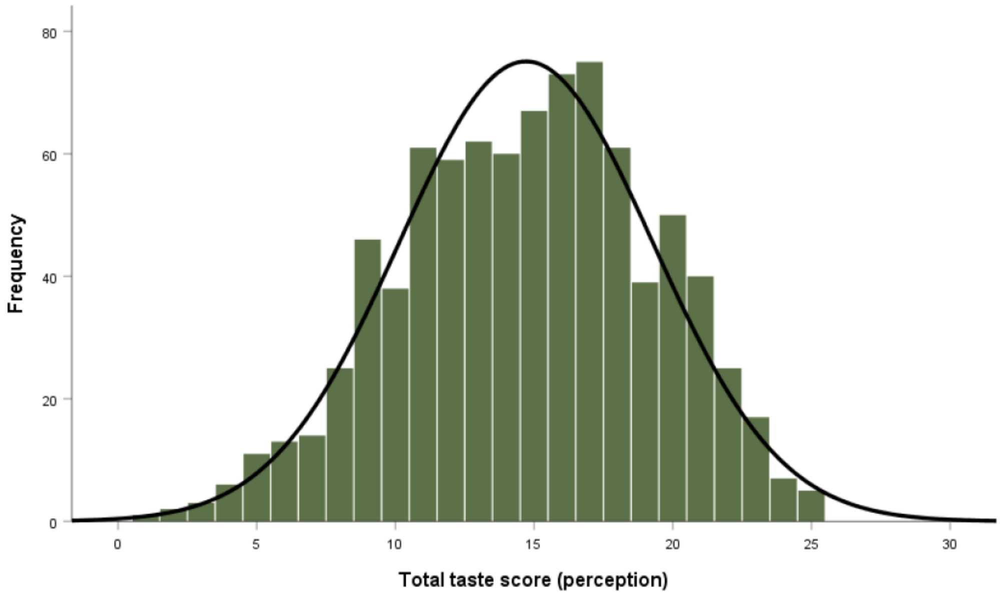

Supplement: Supplementary file 1 [file nutrients-17-00600-s001.zip › nutrients-3443697-supplementary.pdf]
